# Causes of Indigestion

**Published:** 1915-06

**Authors:** 


### ABSTRACTS

---

**Causes of Indigestion,** Douglas VanderHoof, Johns Hopkins Hosp. Bull., May 1915. In an analysis of 1000 cases—indigestion merely meaning complaint of stomach trouble—24.6% were found due to appendicitis, 11.7% to cholecystitis, 25% to various viscera and organs, including 7.1 to kidney lesions. About 35% were really gastric cases, approximately including 10% of gastric ulcer, 10% of gastric neuroses, 5½ of cancer and 10% of miscellaneous organic gastric and associated disease. Note: Personal analyses have shown about 20% of apparently gastric cases, to be really due to the stomach but often only in a direct sense, the gastric lesion or neurosis being ultimately due to some other factor. The figures for cancer and ulcer are about reversed in our experience. Naturally, accidental factors would greatly modify individual experiences and the classification would vary somewhat according to the personal equation of the observer. But practically all statistics of this sort agree in the essential point that gastric symptoms are due, only in a minority of instances to strictly gastric conditions, organic or functional.

